# Positive Effect of Elevated Thawing Rate for Cryopreservation of Human Ovarian Tissue: Transcriptomic Analysis of Fresh and Cryopreserved Cells

**DOI:** 10.3390/ijms252413747

**Published:** 2024-12-23

**Authors:** Qingduo Kong, Plamen Todorov, Cheng Pei, Evgenia Isachenko, Gohar Rahimi, Nina Mallmann-Gottschalk, Volodimir Isachenko

**Affiliations:** 1 Department of Obstetrics and Gynecology, Medical Faculty, Cologne University, 50931 Cologne, Germany; qingduokong@gmail.com (Q.K.); chengpei314@gmail.com (C.P.); evgenia.isachenko@uk-koeln.de (E.I.); gohar.rahimi@amedes-group.com (G.R.); 2 Institute of Biology and Immunology of Reproduction of Bulgarian Academy of Sciences (BAS), 1113 Sofia, Bulgaria; plamen.ivf@gmail.com; 3 Medizinisches Versorgungszentrum AMEDES für IVF- und Pränatalmedizin in Köln GmbH, 50968 Cologne, Germany; 4 Department of Obstetrics and Gynecology, Medical Faculty, Essen University, 45147 Essen, Germany; nina.mallmann-gottschalk@uk-essen.de

**Keywords:** human ovarian tissue, cryopreservation, thawing, RNA sequencing, transcriptomics, differentially expressed genes (DEGs), Kyoto encyclopedia of genes and genomes (KEGG), gene ontology (GO)

## Abstract

Ovarian tissue cryopreservation has been gradually applied. It is essential to elucidate the differences between cryopreserved and fresh ovarian tissue and to refine cryopreservation protocols for improved outcomes. To explore the transcriptomic differences between fresh ovarian tissue and tissue cryopreserved with an elevated thawing rate. Ovarian tissue samples were collected and cryopreserved (frozen and thawed) following RNA sequencing and histological evaluation. Three groups were formed: fresh tissue (Group 1), frozen tissue after quick thawing at 100 °C (Group 2), and frozen tissue after slow thawing at 37 °C (Group 3). KEGG analysis showed that in comparison with Group 1, DEGs in Group 2 were mainly enriched in the cortisol synthesis and ovarian steroidogenesis pathways, and DEGs in the cells of Group 3 were mainly enriched in the ovarian steroidogenesis pathway. GO analysis showed that compared to cells of Group 2, DEGs in Group 3 were primarily enriched in the SRP-dependent co-translational protein targeting pathway and co-translational protein targeting to the membrane. The results were formulated with a minimal difference in the histological evaluation of cells after quick and slow thawed tissue. Cryopreservation of ovarian tissue by the described method does not decrease follicle production but downregulates the ovarian steroidogenesis pathway, reducing estrogen and progesterone secretion. The quick thawing of ovarian tissue increases the proliferation and apoptosis pathways of cells.

## 1. Introduction

With advancements in science and technology, an increasing number of females are becoming aware of fertility preservation options. Fertility preservation is particularly relevant for females undergoing cancer treatment or those diagnosed with premature ovarian insufficiency (POI) [1,2,3]. In addition to oocyte cryopreservation and embryo cryopreservation, ovarian tissue cryopreservation (OTC) offers a more comprehensive approach to preserving ovarian function. This technique has gained significant attention and has become an important strategy for safeguarding female fertility [4,5,6,7]. However, there remains concern regarding whether cryopreserved ovarian tissue can maintain functionality comparable to that of fresh ovarian tissue. OTC has been gradually applied, and so far, more than 200 live births have been achieved, demonstrating its ability to restore fertility by producing functional oocytes [8,9]. Nevertheless, it is essential to elucidate the molecular and functional differences between cryopreserved and fresh ovarian tissue and to refine cryopreservation protocols for improved outcomes.

Currently, the primary techniques for ovarian tissue cryopreservation include conventional slow freezing and vitrification [10,11,12]. Conventional slow freezing is the most widely utilized method for ovarian tissue preservation. This technique systematically decreases the temperature of ovarian tissue to cryogenic levels using a computer-controlled programmable freezer. The critical factor in this method is the precise control of the cooling rate, typically set at 0.3 °C per minute, which minimizes the formation of intracellular and extracellular ice crystals, thereby preserving the integrity of cellular membranes and the overall tissue architecture [13,14]. Vitrification is an ultrarapid freezing technique that utilizes extremely high cooling rates to convert intracellular and extracellular fluids into a glass-like, amorphous solid state, effectively preventing ice crystal formation [15,16]. However, vitrification is less effective for larger tissue volumes, as the temperature gradient between the tissue surface and core can result in asynchronous cooling and potential tissue damage. Consequently, this study selected the more established method of slow freezing.

In addition to the freezing process, the thawing phase is equally critical in ovarian tissue cryopreservation. It is essential to restore the tissue to its pre-freezing state to the greatest extent possible. Primarily, preventing thermal damage to the tissue during thawing is the most important. Conventionally, the tissue is first placed at room temperature for 30 s, followed by immersion in 37 °C warm water until all ice has completely melted. This method minimizes thermal injury to the tissue during thawing. Notably, the first successful live birth resulting from ovarian cryopreservation in Germany employed this thawing method [14,17]. However, it is also imperative to minimize the contact time between the tissue and the cryoprotectant during thawing. Reducing the exposure time to the cryoprotectant while ensuring that the tissue does not experience thermal damage can mitigate the cytotoxic effects of the cryoprotectant on ovarian tissue. During the thawing stage, the cell membrane is particularly vulnerable, and the osmotic pressure across the membrane is unbalanced. Therefore, achieving a rapid transition to the recovery phase is critical for maintaining cellular viability. The study group of the Medical Faculty of Cologne University investigated a method involving the exposure of cryovials to room temperature for 30 s, followed by immersion in a boiling water bath (100 °C) for 60 to 75 s. Moreover, there was precise monitoring of the ice melting, with the immersion time visually controlled: once the ice at the tip of the cryovial was reduced to approximately 1 mm in thickness, the cryovial was removed from the water. This technique minimizes the overall thawing time and the contact time of the tissue with the cryoprotectant while ensuring that thermal damage is avoided.

Consequently, this study used transcriptomic analysis to evaluate the differences between conventional slow freezing and fresh human ovarian tissue, as well as the effects of different thawing rates on ovarian tissue to better optimize ovarian cryopreservation techniques.

## 2. Results

### 2.1. Differential Expression Genes (DEGs)

**Figure 1:**
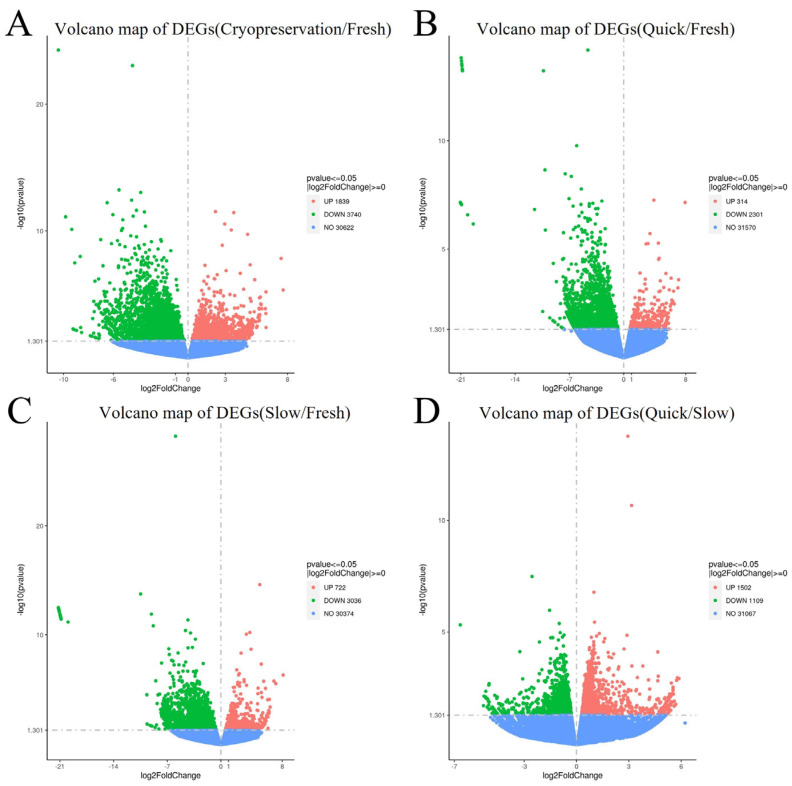
Volcano map of differentially expressed genes (DEGs) (**A**) DEGs volcano map: Group 2 plus Group 3 (cryopreserved ovarian tissue) vs. Group 1 (fresh ovarian tissue). (**B**) DEGs volcano map: Group 2 (frozen ovarian tissue after quick thawing at 100 °C) vs. Group 1 (fresh ovarian tissue). (**C**) DEGs volcano map: Group 3 (frozen ovarian tissue after slow thawing at 37 °C) vs. Group 1 (fresh ovarian tissue). (**D**) DEGs volcano map: Group 2 (frozen ovarian tissue after quick thawing at 100 °C) vs. Group 3 (frozen ovarian tissue after slow thawing at 37 °C).

Volcano plots were initially generated to compare upregulated and downregulated differentially expressed genes across tissues from different groups. In comparison to Group 1 (fresh ovarian tissue), the expression of 1839 genes was upregulated and 3740 genes were downregulated in Group 2 plus Group 3 (Cryopreserved ovarian tissue) as shown in Figure 1A. The expression of 314 genes was upregulated, and 2301 genes were downregulated in Group 2 (frozen ovarian tissue after quick thawing at 100 °C) compared with Group 1 (shown in Figure 1B). At the same time, in Group 3 (frozen ovarian tissue after slow thawing at 37 °C), compared to Group 1, the expression of 722 genes was upregulated, while 3036 genes were downregulated as shown in Figure 1C. Compared to Group 2, Group 3 exhibited upregulation in 1502 genes and downregulation in 1109 genes (shown in Figure 1D).

### 2.2. Kyoto Encyclopedia of Genes and Genomes (KEGG)

**Figure 2:**
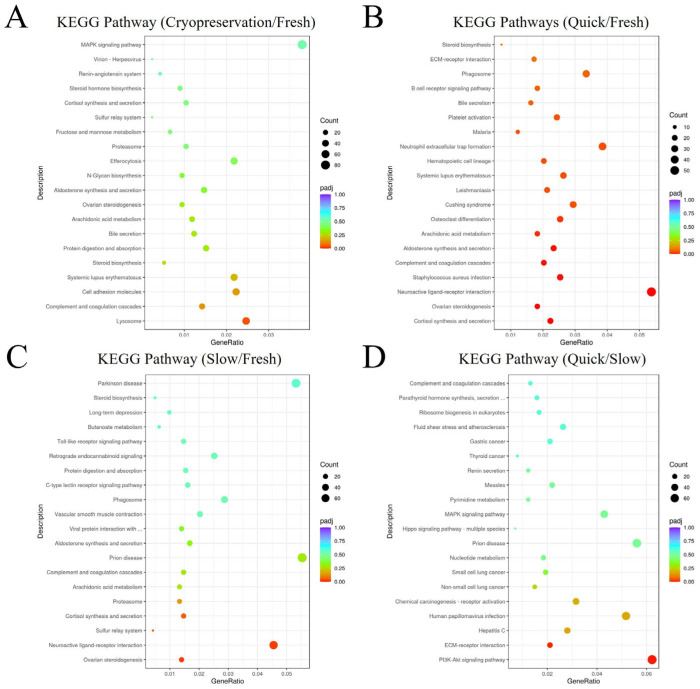
Visualization dot map of Kyoto Encyclopedia of Genes and Genomes (KEGG) pathway analysis (**A**) KEGG pathway analysis: Group 2 plus Group 3 (cryopreserved ovarian tissue) vs. Group 1 (fresh ovarian tissue). (**B**) KEGG pathway analysis: Group 2 (frozen ovarian tissue after quick thawing at 100 °C) vs. Group 1 (fresh ovarian tissue). (**C**) KEGG pathway analysis: Group 3 (frozen ovarian tissue after slow thawing at 37 °C) vs. Group 1 (fresh ovarian tissue). (**D**) KEGG pathway analysis: Group 2 (frozen ovarian tissue after quick thawing at 100 °C) vs. Group 3 (frozen ovarian tissue after slow thawing at 37 °C).

KEGG pathway enrichment analysis based on DEGs was conducted. In comparison to Group 1 (fresh ovarian tissue), DEGs in Group 2 plus Group 3 (cryopreserved ovarian tissue) were mainly enriched in the lysosome pathway as shown in Figure 2A. Compared to Group 1, DEGs in Group 2 (frozen ovarian tissue after quick thawing at 100 °C) were mainly enriched in the cortisol synthesis pathway and ovarian steroidogenesis pathway, while DEGs in Group 3 (frozen ovarian tissue after slow thawing at 37 °C) were mainly enriched in the ovarian steroidogenesis pathway (Figure 2B,C). Compared to Group 2, DEGs in Group 3 were primarily enriched in the PI3K-Akt signaling pathway and ECM-receptor interaction pathway (shown in Figure 2D).

### 2.3. Gene Set Enrichment Analysis (GSEA)

**Figure 3:**
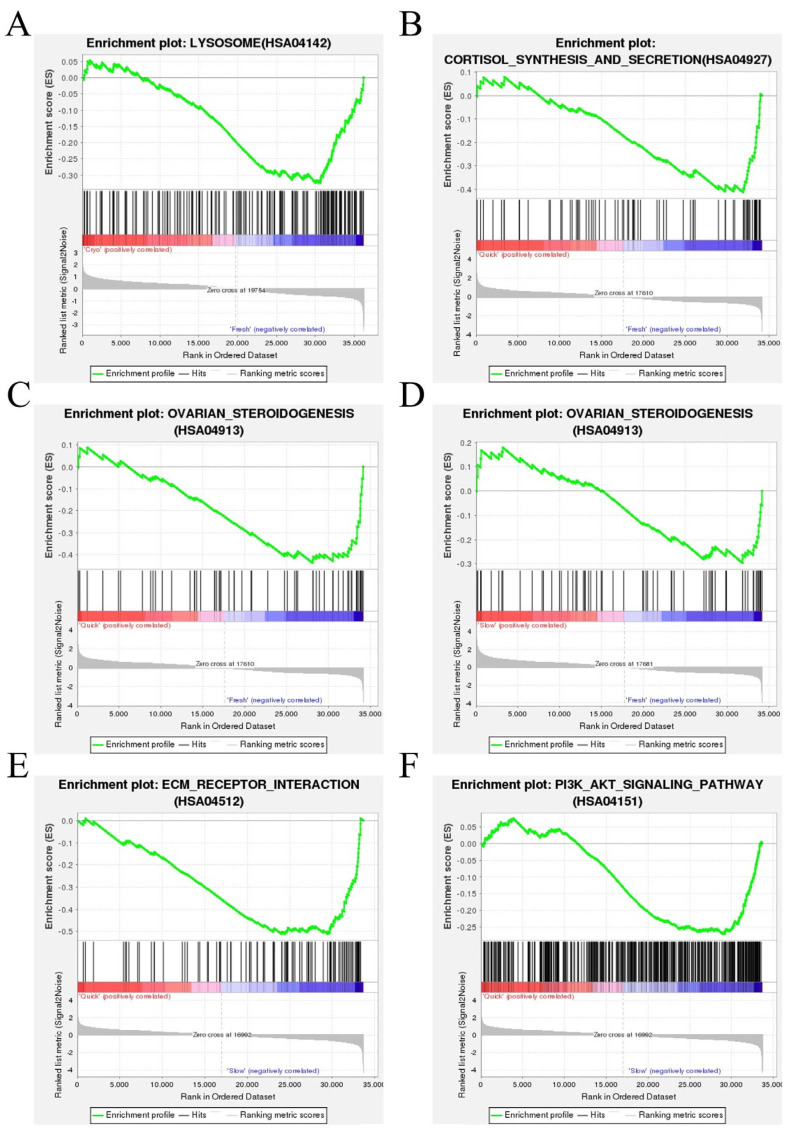
Gene Set Enrichment Analysis (GSEA) based on KEGG pathway data. (**A**) GSEA analysis indicated the lysosome pathway was enriched and downregulated in Group 2 plus Group 3 (cryopreserved ovarian tissue) compared with Group 1 (fresh ovarian tissue). (**B**) GSEA analysis indicated the cortisol synthesis pathway was enriched and downregulated in Group 2 (frozen ovarian tissue after quick thawing at 100 °C) vs. Group 1 (fresh ovarian tissue). (**C**) GSEA analysis indicated the ovarian steroidogenesis pathway was enriched and downregulated in Group 2 (frozen ovarian tissue after quick thawing at 100 °C) vs. Group 1 (fresh ovarian tissue). (**D**) GSEA analysis indicated the ovarian steroidogenesis pathway was enriched and downregulated in Group 3 (frozen ovarian tissue after slow thawing at 37 °C) vs. Group 1 (fresh ovarian tissue). (**E**) GSEA analysis indicated the PI3K-Akt signaling pathway was enriched and downregulated in Group 2 (frozen ovarian tissue after quick thawing at 100 °C) vs. Group 3 (frozen ovarian tissue after slow thawing at 37 °C). (**F**) GSEA analysis indicated the ECM-receptor interaction pathway was enriched and downregulated in Group 2 (frozen ovarian tissue after quick thawing at 100 °C) vs. Group 3 (frozen ovarian tissue after slow thawing at 37 °C).

GSEA is a type of in-depth data analysis based on predefined gene sets in the KEGG database. In comparison to Group 1 (fresh ovarian tissue), the enrichment of genes involved in the lysosome pathway in downregulation in Group 2 plus Group 3 (cryopreserved ovarian tissue) (Figure 3A). It was shown that the expression of the lysosomal pathway in cryopreserved ovarian tissue was significantly reduced compared to that in fresh ovarian tissue. Compared to Group 1, Group 2 (frozen ovarian tissue after quick thawing at 100 °C) showed downregulation of genes involved in the cortisol synthesis pathway (Figure 3B,C) and ovarian steroidogenesis pathway, while Group 3 (frozen ovarian tissue after slow thawing at 37 °C) showed downregulation of genes involved in the ovarian steroidogenesis pathway (Figure 3D). It was shown that both quick-thawing and slow-thawing cryopreserved ovarian tissues exhibited a significant reduction in the expression of the ovarian steroidogenesis pathway compared to fresh ovarian tissue. Compared to Group 3, Group 2 showed downregulation of genes involved in the PI3K-Akt signaling pathway and ECM-receptor interaction pathway (Figure 3E,F). It was shown that quick-thawing cryopreserved ovarian tissues exhibited downregulation of genes in the expression of the PI3K-Akt signaling pathway and ECM-receptor interaction pathway compared to slow-thawing cryopreserved ovarian tissues.

### 2.4. Gene Ontology (GO)

**Figure 4:**
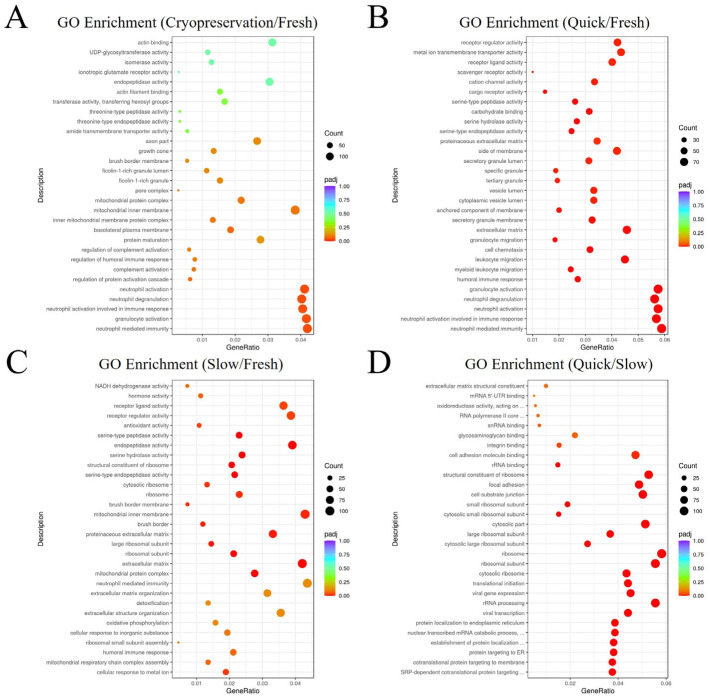
Visualization dot map of Gene Ontology (GO) enrichment analysis. (**A**) GO enrichment analysis: Group 2 plus Group 3 (cryopreserved ovarian tissue) vs. Group 1 (fresh ovarian tissue). (**B**) GO enrichment analysis: Group 2 (frozen ovarian tissue after quick thawing at 100 °C) vs. Group 1 (fresh ovarian tissue). (**C**) GO enrichment analysis: Group 3 (frozen ovarian tissue after slow thawing at 37 °C) vs. Group 1 (fresh ovarian tissue). (**D**) GO enrichment analysis: Group 2 (frozen ovarian tissue after quick thawing at 100 °C) vs. Group 3 (frozen ovarian tissue after slow thawing at 37 °C).

GO analysis based on DEGs was conducted. The analysis covered the functions of the biological process (BP), cellular component (CC), and molecular function (MF). In comparison to Group 1 (fresh ovarian tissue), DEGs in Group 2 plus Group 3 (cryopreserved ovarian tissue) were mainly enriched in neutrophil-mediated immunity and granulocyte activation as shown in Figure 4A. Compared to Group 1, DEGs in Group 2 (frozen ovarian tissue after quick thawing at 100 °C) were mainly enriched in neutrophil-mediated immunity and neutrophil activation involved in immune response, while DEGs in Group 3 (frozen ovarian tissue after slow thawing at 37 °C) were mainly enriched in cellular response to the metal ion and humoral immune response ovarian steroidogenesis pathway (Figure 4B,C). Compared to Group 2, DEGs in Group 3 were primarily enriched in the SRP-dependent co-translational protein targeting pathway and co-translational protein targeting to the membrane (shown in Figure 4D).

### 2.5. Morphology of Ovarian Tissue

**Figure 5:**
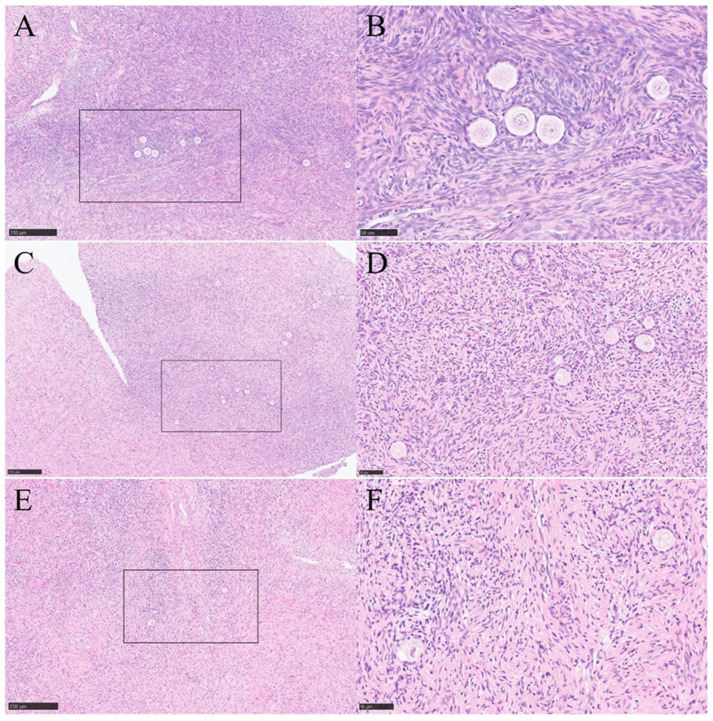
Hematoxylin-Eosin (HE) -staining of fresh ovarian tissue and cryopreserved ovarian tissue. (**A**,**B**) HE-staining of Group 1 (fresh ovarian tissue). (**C**,**D**) HE-staining of Group 2 (frozen ovarian tissue after quick thawing at 100 °C). (**E**,**F**) HE-staining of Group 3 Group 3 (frozen ovarian tissue after slow thawing at 37 °C).Bar for (**A**,**C**,**E**) = 250 µm, bar for (**B**,**D**,**F**) = 50 µm.

Hematoxylin-Eosin (HE)-staining of fresh ovarian tissue is shown in Figure 5A,B. To minimize ice crystal formation during cryopreservation, the ovarian medulla was removed prior to the procedure. The fresh ovarian tissue in this study served as a control for the cryopreserved ovarian tissue, with the ovarian medulla also removed. Consequently, the HE-staining result showed the absence of mature follicles and secondary oocytes, revealing only a limited number of primordial follicles. The primordial follicle was encased by a layer of flat granulosa cells, which were essential for supporting oocyte development and for synthesizing and secreting hormones in the future. Additionally, stromal cells surrounding the follicles provided crucial support and nutrition for their further development. Similar results were also shown in the quick-thawing cryopreserved ovarian tissue in Figure 5C,D and in the slow-thawing cryopreserved ovarian tissue in Figure 5E,F. Clear primordial follicles, along with the surrounding granulosa cells and stromal cells, demonstrated that there was a minimal difference in HE-staining between quick-thawing cryopreserved ovarian tissue, slow-thawing cryopreserved ovarian tissue, and fresh ovarian tissue.

## 3. Discussion

### 3.1. Transcriptomic Differences Between Cryopreserved Ovarian Tissue and Fresh Ovarian Tissue

The primary function of ovarian tissue is the production of oocytes, enabling female fertility. Additionally, it secretes estrogen and progesterone, which are essential for the development and maturation of follicles. In recent years, ovarian cryopreservation technology has advanced significantly, offering improved methods for preserving female fertility. However, no studies have yet investigated the transcriptomic differences between cryopreserved ovarian tissue and fresh ovarian tissue. This study aims to investigate the transcriptomic differences between cryopreserved and fresh ovarian tissue.

Additionally, different thawing rates for cryopreserved ovarian tissue may lead to subtle differences in the ovarian tissue. The traditional thawing method is to leave the cryotube with the fragments of ovarian tissue at room temperature for 30 s and subsequently place it in 37 °C warm water for 1 min until the ice has completely melted. Many studies have demonstrated its effectiveness, and, at the same time, the Cologne study group recommended leaving the cryotube with the fragments of ovarian tissue at room temperature for 30 s and then immersing for a period of 60 to 75 s in a water bath with boiling water (100 °C). When the ice at the tip is thinner than ~1 mm the cryotube should be removed as a quick thawing method. This method aligns with the principle of cryobiology. Any organism cryopreserved using existing cryopreservation techniques should be thawed as quickly as possible to minimize the duration of exposure to cryoprotectants, thereby reducing excessive dehydration and osmotic imbalance, and minimizing cell damage. However, the effectiveness of this quick thawing method requires further investigation. This study aims to explore whether different thawing rates produce significant differences in transcriptomic profiles compared to fresh ovarian tissue.

#### 3.1.1. Differentially Expressed Genes (DEGs)

Identifying DEGs is a fundamental step in transcriptomic analysis, aimed at understanding the extent to which gene expression differences respond to specific conditions changes. The results from our experiment indicate significant differences in gene expression between cryopreserved ovarian tissue and fresh ovarian tissue, with over 5000 differentially expressed genes. Furthermore, more than 2000 genes exhibited significant changes between quick thawing cryopreserved ovarian tissue and fresh ovarian tissue, while over 3000 genes were identified as differentially expressed between slow-thawing cryopreserved ovarian tissue and fresh ovarian tissue. It demonstrates that, regardless of the thawing rate used, significant transcriptomic changes exist between cryopreserved ovarian tissue and fresh ovarian tissue.

In the comparison between cryopreserved ovarian tissue and fresh ovarian tissue, the genes that were significantly upregulated in cryopreserved ovarian tissue included FOSB and JUN. FOXB is a member of the Forkhead Box (FOX) family of transcription factors, while JUN forms heterodimers with FOSB to create the AP-1 transcription factor. This factor plays a critical role in important signaling pathways that regulate gene expression and which are involved in various physiological processes, including cell proliferation, apoptosis, stress response, and inflammation [18,19]. It suggests that a complex interplay of proliferation and apoptosis processes is implicated in the cryopreservation process. The significantly downregulated gene in cryopreserved ovarian tissue included DHCR4, which is closely related to cholesterol synthesis [20,21]. Besides oocyte production, the primary function of ovarian tissue is the synthesis of estrogen and progesterone, both of which are vital steroid hormones. Consequently, cryopreservation may impact the secretion of these hormones. Given the extensive changes observed in gene expression, we conducted pathway enrichment analysis to identify specific pathways that exhibit significant alterations between cryopreserved ovarian tissue and fresh ovarian tissue.

#### 3.1.2. Kyoto Encyclopedia of Genes and Genomes (KEGG) and Gene Set Enrichment Analysis (GSEA) Analysis

We found that only one pathway exhibited significant changes in KEGG analysis between cryopreserved ovarian tissue and fresh ovarian tissue, specifically the lysosomal pathway. Combined with GSEA, it was further confirmed that the lysosomal pathway is downregulated in the cryopreserved group. The lysosomal pathway is a crucial degradation pathway within cells, primarily responsible for processing cellular waste. This pathway plays an essential role in maintaining cell homeostasis, energy metabolism, signal transduction, and immune responses. Downregulation of the lysosomal pathway impairs the cell’s ability to respond to stress, resulting in diminished lysosomal function, a reduced number of lysosomes, decreased enzyme activity, accumulation of toxic substances within the cells, and potential induction of cell death [22,23,24]. Consequently, during the cryopreservation process, cells may activate stress responses, and the downregulation of the lysosomal pathway diminishes the tissues’ ability to manage these stressors, leading to the accumulation of endotoxins and promoting cellular apoptosis. When faced with the downregulation of the lysosomal pathway, it is necessary to implement appropriate intervention measures during the cryopreservation process to enhance the characteristics of cryopreserved ovarian tissue to resemble those of fresh ovarian tissue; the cryopreserved ovarian tissue may be better than before. The studies indicate that the degradative function of lysosomes can be enhanced by activating the autophagy pathway [25]. Furthermore, the downregulation of the lysosomal function is frequently associated with elevated oxidative stress in cells [26]. Supplementation with antioxidants may alleviate oxidative stress-induced damage to lysosomes and indirectly enhance lysosomal function. More research is necessary to investigate the potential incorporation of these substances into the cryopreservation process to more effectively preserve the characteristics of cryopreserved ovarian tissue.

We found that in KEGG pathways analysis that changed significantly between quick-thawing cryopreserved ovarian tissue and fresh ovarian tissue were the cortisol synthesis and secretion pathway and the ovarian steroidogenesis pathway. At the same time, slow-thawing cryopreserved ovarian tissue and fresh ovarian tissue changed significantly, and this was also on the ovarian steroidogenesis pathway. Combined with GSEA, it can be confirmed further that the ovarian steroidogenesis pathway is downregulated in both the quick-thawing cryopreserved ovarian tissue group and the slow-thawing cryopreserved ovarian tissue group compared with fresh ovarian tissue. The ovarian steroidogenesis pathway uses cholesterol as a raw material in theca cells and granulosa cells of the follicles and is catalyzed by a series of enzymes to gradually generate different steroid hormones, mainly estrogen and progesterone [27,28]. The primary goal of ovarian cryopreservation is to preserve ovarian function, which encompasses not only the production of oocytes but also the synthesis of estrogen and progesterone. However, transcriptomic analysis has revealed that the ovarian steroidogenesis pathway is significantly downregulated in the cryopreserved group, leading to a reduction in the synthesis of estrogen and progesterone. Considering the downregulation of this pathway, it should be better to give appropriate intervention measures during the cryopreservation process, such as adding follicle-stimulating hormone (FSH) and luteinizing hormone (LH) in the cryopreservation process to promote the synthesis of estrogen and progesterone. Following cryopreservation and thawing, in vitro culture can be conducted, during which FSH and LH can be supplemented to promote the synthesis of estrogen and progesterone. Currently, FSH and LH are also incorporated into in vitro culture to further enhance the growth and development of ovarian cells. This study demonstrates that the ovarian steroidogenesis pathway is compromised during the cryopreservation process. Adding FSH and LH serves not only to promote the growth and development of ovarian cells but also to compensate for the damage incurred due to the downregulation of the ovarian steroidogenesis pathway during the cryopreservation process.

#### 3.1.3. Gene Ontology (GO) Analysis

It can be found that the pathways with significant changes in GO analysis between cryopreserved ovarian tissue and fresh ovarian tissue included inflammation-related pathways, such as the neutrophil-mediated immune response pathway and the granulocyte activation pathway. During the cryopreservation process, cells are exposed to environmental changes that trigger stress responses. Notably, inflammation-related responses differ between quick-thawing cryopreserved ovarian tissue and fresh ovarian tissue, as well as between slow-thawing cryopreserved ovarian tissue and fresh ovarian tissue. These changes are linked to the stress state of the cells and exhibit minimal correlation with ovarian cell development, fertility, proliferation, or apoptosis.

### 3.2. Transcriptomic Differences Between Quick-Thawing Cryopreserved Ovarian Tissue and Slow-Thawing Cryopreserved Ovarian Tissue

The differences between cryopreserved ovarian tissues with two thawing rates and fresh ovarian tissues are obvious. However, the distinctions between the two thawing rates themselves remain unclear. For instance, the results of hematoxylin and eosin (HE) staining indicated that both thawing rates can generate viable follicles. However, whether quick thawing results in less damage to ovarian tissues compared to slow thawing requires further investigation. Studies have shown that quick thawing may offer several advantages. Firstly, it can reduce ice crystal formation by effectively preventing and minimizing the regrowth of ice crystals during the thawing process. In contrast, during slow thawing, residual cryoprotectants or water molecules may recrystallize, leading to the formation of ice crystals. These ice crystals can inflict physical damage to both intracellular and extracellular structures, including disrupting cell membranes and damaging organelles. Secondly, quick thawing helps preserve cell structure and function by maintaining the integrity of the cell membrane in ovarian tissue and protecting the functions of key cell types, such as follicles and granulosa cells. Prolonged exposure of cells to cryoprotectants during slow thawing can increase their toxicity, leading to cell membrane damage, metabolic disorders, and even cell death. Additionally, slow thawing may result in incomplete or unstable phase transitions of the cell membrane, particularly in the presence of cryoprotectants, which can further compromise membrane integrity and make it more fragile. Finally, quick thawing facilitates better osmotic pressure balance between the intracellular and extracellular environments. Prolonged resuscitation times can exacerbate damage to the membrane structure, negatively impacting cell integrity and function. Fluctuations in temperature during the resuscitation process, coupled with the removal of cryoprotectants, can disrupt osmotic pressure equilibrium. If the recovery period is excessively lengthy, osmotic pressure may not normalize swiftly, leading to cell dehydration or excessive hydration. This imbalance can cause cells to either swell or shrink, ultimately affecting their survival and functionality [29,30,31,32]. This study aims to investigate the transcriptomic differences between quick and slow thawing rates.

#### 3.2.1. Differentially Expressed Genes (DEGs)

The results indicated that there are numerous differentially expressed genes in quick- and slow-thawing cryopreserved ovarian tissue, with over 2000 genes showing significant changes. This finding confirms that there are distinct transcriptomic differences between quick- and slow-thawing cryopreserved ovarian tissue. In the comparison between the quick-thawing group and the slow-thawing group, the gene that was significantly upregulated in the quick-thawing group was GADD45B, which is a member of the GADD45 family. Members of the GADD45 family are involved in various biological processes, including the cellular stress response, the regulation of proliferation, and DNA repair. Notably, GADD45B is particularly sensitive to a range of stressors and serves as a crucial regulator of cellular responses to environmental changes [33,34,35]. This finding indicates that the cellular stress response in the quick-thawing group is more pronounced. When the cellular stress response is elevated, it can elicit a series of complex biological effects, both beneficial and detrimental. On the positive side, the stress response can activate cellular repair mechanisms, such as DNA repair, protein folding, and degradation, thereby aiding in the restoration of normal cellular function. It also promotes cell proliferation, differentiation, and immune activity. Conversely, an excessive stress response can result in the overproduction of reactive oxygen species (ROS), leading to oxidative damage that affects cell membranes, DNA, and proteins, as well as promoting apoptosis. Therefore, while an enhanced cellular stress response may confer protective effects, such as tissue repair and immune enhancement, excessive stress can cause cellular damage and apoptosis. Consequently, although the quick-thawing group elicited an increased stress response, further investigation is needed to determine whether this ultimately leads to enhanced cell proliferation or increased apoptosis. Because the number of genes changed was too high, we conducted pathway enrichment analysis to identify the specific pathways that exhibited significant changes between quick- and slow-thawing cryopreserved ovarian tissue.

#### 3.2.2. Kyoto Encyclopedia of Genes and Genomes (KEGG) and Gene Set Enrichment Analysis (GSEA) Analysis

It was identified in KEGG analysis between quick- and slow-thawing cryopreserved ovarian tissue that the pathways with significant changes included the PI3K-Akt signaling pathway and ECM-receptor interaction pathway. Combined with GSEA, it can be confidently confirmed that compared to slow-thawing cryopreserved ovarian tissue, the PI3K-Akt signaling pathway and ECM-receptor interaction pathway in the quick-thawing cryopreserved ovarian tissue were significantly downregulated. The PI3K-Akt signaling pathway plays a critical role in various biological processes, including cell growth, proliferation, survival, migration, and metabolism. It primarily promotes cell cycle progression, enhances cell proliferation, and increases cell survival by inhibiting apoptotic pathways [36,37]. The significant downregulation of this pathway may suggest that cell survival in the quick-thawing group is markedly lower than in the slow-thawing one. The ECM-receptor interaction pathway is crucial for cell signaling, proliferation, differentiation, and migration. Downregulation of this pathway may lead to decreased adhesion between cells and the extracellular matrix, negatively impacting cell morphology, migration, and proliferation [38,39,40]. This further substantiates that morphological changes in the quick-thawing group promote apoptosis. Consequently, it indicates that a series of apoptotic processes occurred in the quick-thawing group following the stress response.

#### 3.2.3. Gene Ontology (GO) Analysis

It was identified in GO analysis between quick- and slow-thawing cryopreserved ovarian tissue that the pathways with significant changes included the SRP-dependent co-translational protein targeting pathway and co-translational protein targeting to the membrane. The SRP-dependent co-translational protein targeting pathway is a critical protein transport mechanism within the cell, primarily responsible for directing secretory proteins, membrane proteins, and proteins destined for organelles to the endoplasmic reticulum (ER) during synthesis. This process ensures that newly synthesized polypeptides are accurately directed as they are produced on ribosomes. Under stress conditions, upregulation of the SRP pathway may help cells synthesize and transport stress-related proteins more effectively. By enhancing the protein-processing capacity of the ER, upregulation of the SRP pathway can improve cell survival and reduce cell death. The co-translational targeting protein mechanism plays an important role in intracellular protein synthesis and transport. Through effective signal recognition and transport processes, cells can ensure the correct folding and function of proteins, thereby maintaining the normal operation and physiological function of cells. The upregulation of this pathway, together with the SRP pathway, promotes protein synthesis, improves cell survival, and reduces cell death [41,42,43]. Consequently, cells in the quick-thawing group exhibited heightened stress responses. Activation of the SRP-dependent pathway and the co-translational targeting pathway can enhance cell survival and mitigate cell death. In the KEGG pathway enrichment analysis, it was determined that the quick-thawing group facilitated the apoptotic process via the PI3K-Akt signaling pathway and ECM-receptor interaction pathway following the stress response. However, the GO results indicated that the quick-thawing group also promoted cell survival through the SRP-dependent pathway and the co-translational targeting pathway in response to stress. Thus, following the stress response, quick-thawing cryopreserved ovarian tissue exhibited both pro-apoptotic and pro-survival activities. Further investigation is required to determine which pathway has a stronger influence.

### 3.3. Limitations

This study aims to explore the transcriptomic differences between fresh ovarian tissue and tissue cryopreserved with an elevated thawing rate. Our results indicate that cryopreservation of ovarian tissue by the described method does not decrease follicle production but downregulates the ovarian steroidogenesis pathway, reducing estrogen and progesterone secretion. Quick thawing of ovarian tissue increases the proliferation and apoptosis pathways of cells. The main limitation of our study is the small number of ovarian tissue samples. As human ovarian tissue is difficult to obtain due to ethical and clinical constraints, this has restricted the number of experimental samples for our study. Further studies are required to validate and expand upon the conclusions presented in this article.

## 4. Materials and Methods

### 4.1. Design of Experiments

**Figure 6:**
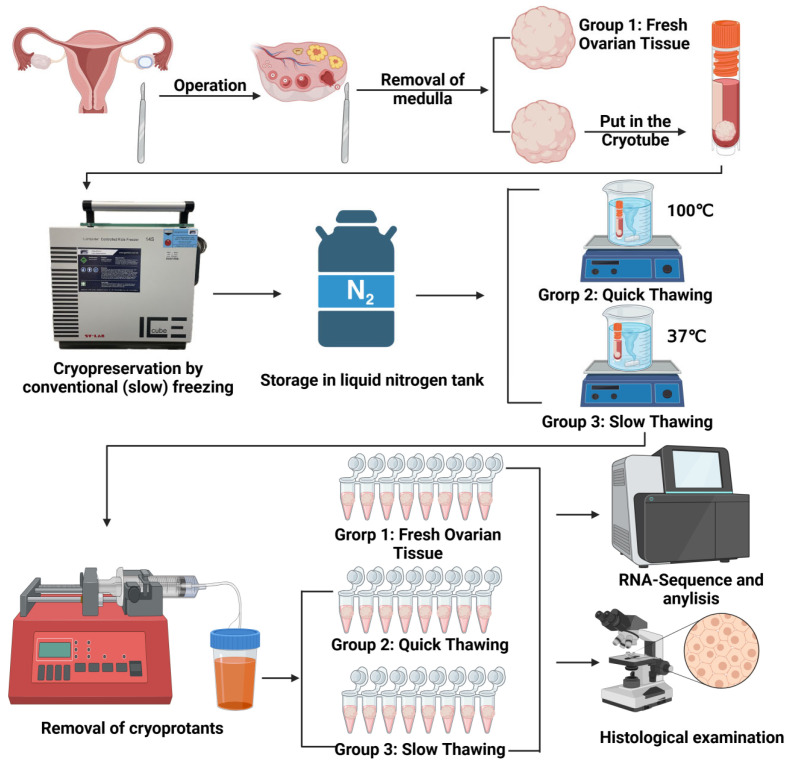
Design of experiments.

A total of 9 human ovarian tissue samples was collected and divided into 3 groups. The technology in our studies was developed for tumor patients. The ovarian tissue fragments used for the experiments were obtained from patients involved in a fertility treatment program. They were all diagnosed with tumors (including Hodgkin’s Lymphoma, Non-Hodgkin’s Lymphoma, and Chronic Myeloid Leukemia). Informed consent was obtained from patients whose tissue was collected for this study. Ovarian tissue fragments were obtained from 3 patients aged 18, 26 and 27 (median age 23.7 years). Three tissue fragments from each patient were used for experiments. Three groups were formed: fresh tissue (Group 1), frozen tissue after quick thawing at 100 °C (Group 2), and frozen tissue after slow thawing at 37 °C (Group 3). Three samples were used in each group (Figure 6).

### 4.2. Collection and Cryopreservation of Ovarian Tissue

The study was conducted following the Declaration of Helsinki and approved by the Institutional Ethics Committee of Cologne University (applications 999,184 and 13-147) and by the Bulgarian Ethics Committee. Informed consent was obtained from patients whose ovarian tissue was collected for this study. All chemicals were obtained from Sigma (Sigma Chemical Co., St. Louis, MO, USA) unless otherwise stated.

Cryopreservation of ovarian tissue was conducted following our previously published protocols [44,45,46,47]. On the day of freezing, ovarian tissue pieces were placed at room temperature in 20 mL of the freezing medium, consisting of basal medium supplemented with 6% dimethyl sulfoxide (DMSO), 6% ethylene glycol, and 0.15 M sucrose. The tissue pieces were then transferred into standard 5 mL cryo-vials (Thermo Fisher Scientific, Rochester, NY, USA), prefilled with the freezing medium, and frozen using the IceCube 14S freezer (SyLab, Neupurkersdorf, Austria). The cryopreservation protocol was as follows: (1) the starting temperature was −6 °C; (2) samples were cooled from −6 °C to −34 °C at a rate of 0.3 °C/min; and (3) at −34 °C, the cryo-vials were plunged into liquid nitrogen. The freezing protocol also included an auto-seeding step at −6 °C.

Quick thawing: Tissue thawing was achieved by placing the cryovials at room temperature for 30 s and then immersing them in a 100 °C (boiling water) water bath for 60 s and draining the vial contents into the solution to remove the cryoprotectant. The exposure time in boiling water was visually controlled by the presence of ice in the culture medium; the vials were removed from the boiling water once the ice reached a size of 2 to 1 mm, at which point the final temperature of the culture medium was between 4 and 10 °C. Within 5 to 10 s of thawing, the tissue fragments in the cryovials were drained into 10 mL of thawing solution (basal medium containing 0.5 M sucrose) in a 100 mL specimen container (Sarstedt, Neumbrecht, Germany). After exposure of the tissue to sucrose for 15 min, stepwise rehydration of the cells was performed as previously reported.

Slow thawing: This thawing method is the same as the quick-thawing method described above, except that the cryovials were immersed in a 37 °C water bath for 1 min to thaw the tissue until the ice completely melted.

### 4.3. Sequencing and Data Extraction

Each sample of ovarian tissue was used for RNA extraction with the Trizol method. RNA integrity was assessed using the Bioanalyzer 2100 system (Agilent Technologies, Santa Clara, CA, USA). Messenger RNA was purified from total RNA using poly-T oligo-attached magnetic beads. After fragmentation, the first strand of cDNA was synthesized using random hexamer primers. Then the second strand of cDNA was synthesized using dUTP, instead of dTTP. The directional library was ready after end repair, A-tailing, adapter ligation, size selection, amplification, and purification. The library was checked with Qubit and real-time PCR for quantification and bioanalyzer for size distribution detection. After library quality control, different libraries were pooled based on the effective concentration and targeted data amount, then subjected to Illumina sequencing. The basic principle of sequencing is “Sequencing by Synthesis”, where fluorescently labeled dNTPs, DNA polymerase, and adapter primers are added to the sequencing flow cell for amplification. As each sequencing cluster extends its complementary strand, the addition of each fluorescently labeled dNTP releases a corresponding fluorescence signal. The sequencer captures these fluorescence signals and converts them into sequencing peaks through computer software, thereby obtaining the sequence information of the target fragment. Reference genome and gene model annotation files were downloaded from the genome website directly. The index of the reference genome was built using Hisat2 v2.0.5, and paired-end clean reads were aligned to the reference genome using Hisat2 v2.0.5. We selected Hisat2 as the mapping tool for Hisat2 can generate a database of splice junctions based on the gene model annotation file and thus obtain a better mapping result than other non-splice mapping tools. The mapped reads of each sample were assembled by StringTie (v1.3.3b) in a reference-based approach. StringTie uses a novel network flow algorithm as well as an optional de novo assembly step to assemble and quantitate full-length transcripts representing multiple splice variants for each gene locus.

### 4.4. Differential Expression Analysis

For DESeq2 with biological replicates, differential expression analysis for two conditions/groups was performed using the DESeq2 R package (1.20.0). DESeq2 provides statistical programs for determining differential expression in digital gene expression data using models based on negative binomial distribution. The resulting *p*-value is adjusted using Benjamini and Hochberg’s methods to control the error discovery rate. The corrected *p*-value ≤ 0.05 and |log2(foldchange)| ≥ 1 was set as the threshold of significant differential expression.

### 4.5. GO and KEGG Enrichment Analysis of Differentially Expressed Genes

GO enrichment analysis of differentially expressed genes was implemented by the clusterProfiler R package, in which gene length bias was corrected. GO terms with corrected *p*-values less than 0.05 were considered significantly enriched by differentially expressed genes. KEGG is a database resource for understanding high-level functions and utilities of the biological system, such as the cell, the organism, and the ecosystem, from molecular-level information, especially large-scale molecular datasets generated by genome sequencing and other high-throughput experimental technologies (http://www.genome.jp/kegg/). We used the cluster profile R package to test the statistical enrichment of differential expression genes in KEGG pathways.

### 4.6. Gene Set Enrichment Analysis

Gene Set Enrichment Analysis (GSEA) is a computational approach to determine if a predefined Gene Set can show a significant consistent difference between two biological states. The genes were ranked according to the degree of differential expression in the two samples, and then the predefined Gene Set was tested to see if they were enriched at the top or bottom of the list. Gene Set Enrichment Analysis can include subtle expression changes. We use the local version of the GSEA analysis tool http://www.broadinstitute.org/gsea/index.jsp, GO, and KEGG data sets were used for GSEA independently.

### 4.7. Histological Analysis

Ovarian tissues were fixed in 3.5% paraformaldehyde for 24 h at 4 °C and then embedded in paraffin wax. Sections of 4 µm thickness were cut, with every 10th section mounted on glass slides and stained using hematoxylin and eosin. Morphological analysis of tissue development and viability was performed under a Nikon Diaphot 300 microscope at 200× and 400× magnification.

## 5. Conclusions

Cryopreservation of ovarian tissue by the described method does not decrease follicle production but downregulates the ovarian steroidogenesis pathway, reducing estrogen and progesterone secretion. Quick thawing of ovarian tissue increases the proliferation and apoptosis pathways of cells.

## Data Availability

The raw data of RNA-seq can be downloaded at “Sequence read archive” on National Center for Biotechnology Information (https://dataview.ncbi.nlm.nih.gov/object/PRJNA1188025?reviewer=or17fgchi8hki9n00bv4e3n1b3, accessed on 12 December 2024).

## References

[1] Oktay K. (2018). Fertility preservation in patients with cancer: ASCO clinical practice guideline update. J. Clin. Oncol..

[2] Christianson M.S., Oktay K. (2019). Advances in fertility-preservation surgery: Navigating new frontiers. Fertil. Steril..

[3] Antunes M.B., Cardeal S.P., Magalhães M., Vale-Fernandes E., Barreiro M., Sá R., Sousa M. (2023). Preservation of fertility in female patients with hematologic diseases. Blood Rev..

[4] Vuković P., Kasum M., Orešković D., Čehić E., Raguž J., Elezaj S., Beketić-Orešković L. (2019). Importance of ovarian tissue cryopreservation in fertility preservation and anti-aging treatment. Gynecol. Endocrinol. Off. J. Int. Soc. Gynecol. Endocrinol..

[5] Fraison E., Huberlant S., Labrune E., Cavalieri M., Montagut M., Brugnon F., Courbiere B. (2023). Live birth rate after female fertility preservation for cancer or haematopoietic stem cell transplantation: A systematic review and meta-analysis of the three main techniques; embryo, oocyte and ovarian tissue cryopreservation. Hum. Reprod..

[6] Dolmans M.M., Donnez J., Cacciottola L. (2021). Fertility Preservation: The Challenge of Freezing and Transplanting Ovarian Tissue. Trends Mol. Med..

[7] Dolmans M.M., Donnez J. (2021). Fertility preservation in women for medical and social reasons: Oocytes vs ovarian tissue. Best Pract. Res. Clin. Obstet. Gynaecol..

[8] Donnez J., Squifflet J., Jadoul P., Demylle D., Cheron A.C., Van Langendonckt A., Dolmans M.M. (2011). Pregnancy and live birth after autotransplantation of frozen-thawed ovarian tissue in a patient with metastatic disease undergoing chemotherapy and hematopoietic stem cell transplantation. Fertil. Steril..

[9] Roux C., Amiot C., Agnani G., Aubard Y., Rohrlich P.S., Piver P. (2010). Live birth after ovarian tissue autograft in a patient with sickle cell disease treated by allogeneic bone marrow transplantation. Fertil. Steril..

[10] Zhou X.H. (2016). Comparison of vitrification and conventional slow freezing for cryopreservation of ovarian tissue with respect to the number of intact primordial follicles: A meta-analysis. Medicine.

[11] Kometas M., Christman G.M., Kramer J., Rhoton-Vlasak A. (2021). Methods of Ovarian Tissue Cryopreservation: Is Vitrification Superior to Slow Freezing?-Ovarian Tissue Freezing Methods. Reprod. Sci..

[12] Herraiz S. (2014). Improving ovarian tissue cryopreservation for oncologic patients: Slow freezing versus vitrification, effect of different procedures and devices. Fertil. Steril..

[13] Vanni V.S., De Lorenzo R., Privitera L., Canti V., Viganò P., Rovere-Querini P. (2019). Safety of fertility treatments in women with systemic autoimmune diseases (SADs). Expert Opin. Drug Saf..

[14] Isachenko V., Dittrich R., Keck G., Isachenko E., Rahimi G., van der Ven H., Montag M., Hoffmann I., Müller A., Distler W. (2012). Cryopreservation of Ovarian Tissue: Detailed Description of Methods for Transport, Freezing and Thawing. Geburtshilfe Frauenheilkd.

[15] Rahimi G. (2009). Apoptosis in human ovarian tissue after conventional freezing or vitrification and xenotransplantation. CryoLetters.

[16] Amorim C.A., Curaba M., Van Langendonckt A., Dolmans M.M., Donnez J. (2011). Vitrification as an alternative means of cryopreserving ovarian tissue. Reprod. Biomed. Online.

[17] Müller A., Keller K., Wacker J., Dittrich R., Keck G., Montag M., Van der Ven H., Wachter D., Beckmann M.W., Distler W. (2012). Retransplantation of cryopreserved ovarian tissue: The first live birth in Germany. Dtsch. Ärzteblatt Int..

[18] Kouzarides T., Ziff E. (1989). Leucine zippers of fos, jun and GCN4 dictate dimerization specificity and thereby control DNA binding. Nature.

[19] Gazon H., Barbeau B., Mesnard J.M., Peloponese J.M. (2017). Hijacking of the AP-1 Signaling Pathway during Development of ATL. Front. Microbiol..

[20] Filipovic I., Buddecke E. (1987). Calmodulin antagonists suppress cholesterol synthesis by inhibiting sterol delta 24 reductase. Lipids.

[21] Fernández C., Suárez Y., Ferruelo A.J., Gómez-Coronado D., Lasunción M.A. (2002). Inhibition of cholesterol biosynthesis by Delta22-unsaturated phytosterols via competitive inhibition of sterol Delta24-reductase in mammalian cells. Biochem. J..

[22] Tan J.X., Finkel T. (2022). A phosphoinositide signalling pathway mediates rapid lysosomal repair. Nature.

[23] Wang F., Gómez-Sintes R., Boya P. (2018). Lysosomal membrane permeabilization and cell death. Traffic.

[24] Yang C., Wang X. (2021). Lysosome biogenesis: Regulation and functions. J. Cell Biol..

[25] Levine B., Kroemer G. (2019). Biological Functions of Autophagy Genes: A Disease Perspective. Cell.

[26] Pellegrini F.R., De Martino S., Fianco G., Ventura I., Valente D., Fiore M., Trisciuoglio D., Degrassi F. (2023). Blockage of autophagosome-lysosome fusion through SNAP29 O-GlcNAcylation promotes apoptosis via ROS production. Autophagy.

[27] Li L., Shi X., Shi Y., Wang Z. (2021). The Signaling Pathways Involved in Ovarian Follicle Development. Front. Physiol..

[28] Wood J.R., Strauss J.F. (2002). Multiple signal transduction pathways regulate ovarian steroidogenesis. Rev. Endocr. Metab. Disord..

[29] Hagedorn J., Alkurdi G., Barker S.A., Brunotte R., Deeb T., Hubenia O., Khayyat D., Leal-Marin S., Rittinghaus T., Glasmacher B. (2023). The technology in cryotechnology. CryoLetters.

[30] Whaley D., Damyar K., Witek R.P., Mendoza A., Alexander M., Lakey J.R. (2021). Cryopreservation: An Overview of Principles and Cell-Specific Considerations. Cell Transplant..

[31] Pegg D.E. (2007). Principles of cryopreservation. Methods Mol. Biol..

[32] Baust J.M., Campbell L.H., Harbell J.W. (2017). Best practices for cryopreserving, thawing, recovering, and assessing cells. Vitr. Cell. Dev. Biol. Anim..

[33] Liebermann D.A., Hoffman B. (2007). Gadd45 in the response of hematopoietic cells to genotoxic stress. Blood Cells Mol. Dis..

[34] Palomer X., Salvador J.M., Griñán-Ferré C., Barroso E., Pallàs M., Vázquez-Carrera M. (2024). GADD45A: With or without you. Med. Res. Rev..

[35] Hoffman B., Liebermann D.A. (2009). Gadd45 modulation of intrinsic and extrinsic stress responses in myeloid cells. J. Cell Physiol..

[36] Fresno Vara J.A., Casado E., de Castro J., Cejas P., Belda-Iniesta C., González-Barón M. (2004). PI3K/Akt signalling pathway and cancer. Cancer Treat. Rev..

[37] Jafari M., Ghadami E., Dadkhah T., Akhavan-Niaki H. (2019). PI3k/AKT signaling pathway: Erythropoiesis and beyond. J. Cell Physiol..

[38] Horta C.A., Doan K., Yang J. (2023). Mechanotransduction pathways in regulating epithelial-mesenchymal plasticity. Curr. Opin. Cell Biol..

[39] Levi N., Papismadov N., Solomonov I., Sagi I., Krizhanovsky V. (2020). The ECM path of senescence in aging: Components and modifiers. FEBS J..

[40] Rosso F., Giordano A., Barbarisi M., Barbarisi A. (2004). From cell-ECM interactions to tissue engineering. J. Cell Physiol..

[41] Jung S.J., Kim H. (2021). Emerging View on the Molecular Functions of Sec62 and Sec63 in Protein Translocation. Int. J. Mol. Sci..

[42] Colombo S.F., Fasana E. (2011). Mechanisms of insertion of tail-anchored proteins into the membrane of the endoplasmic reticulum. Curr. Protein Pept. Sci..

[43] Mehlhorn D.G., Asseck L.Y., Grefen C. (2021). Looking for a safe haven: Tail-anchored proteins and their membrane insertion pathways. Plant Physiol..

[44] Zhou Y., Wang W., Todorov P., Pei C., Isachenko E., Rahimi G., Mallmann P., Nawroth F., Isachenko V. (2023). RNA Transcripts in Human Ovarian Cells: Two-Time Cryopreservation Does Not Affect Developmental Potential. Int. J. Mol. Sci..

[45] Isachenko V., Morgenstern B., Todorov P., Isachenko E., Mallmann P., Hanstein B., Rahimi G. (2020). Patient with ovarian insufficiency: Baby born after anticancer therapy and re-transplantation of cryopreserved ovarian tissue. J. Ovarian Res..

[46] Isachenko V., Mallmann P., Petrunkina A.M., Rahimi G., Nawroth F., Hancke K., Felberbaum R., Genze F., Damjanoski I., Isachenko E. (2012). Comparison of in vitro- and chorioallantoic membrane (CAM)-culture systems for cryopreserved medulla-contained human ovarian tissue. PLoS ONE.

[47] Isachenko V., Todorov P., Isachenko E., Rahimi G., Hanstein B., Salama M., Mallmann P., Tchorbanov A., Hardiman P., Getreu N. (2016). Cryopreservation and xenografting of human ovarian fragments: Medulla decreases the phosphatidylserine translocation rate. Reprod. Biol. Endocrinol. RBE.

